# An Application for Skin Macules Characterization Based on a 3-Stage Image-Processing Algorithm for Patients with Diabetes

**DOI:** 10.1155/2018/9397105

**Published:** 2018-12-16

**Authors:** Cinthya Lourdes Toledo Peral, Francisco José Ramos Becerril, Gabriel Vega Martínez, Arturo Vera Hernández, Lorenzo Leija Salas, Josefina Gutiérrez Martínez

**Affiliations:** ^1^División de Investigación en Ingeniería Médica, Instituto Nacional de Rehabilitación “Luis Guillermo Ibarra Ibarra”, Calz. México–Xochimilco No. 289, Col. Arenal de Guadalupe, Tlalpan, C.P. 14389 Ciudad de México, Mexico; ^2^Servicio de Rehabilitación Cardiaca, Instituto Nacional de Rehabilitación “Luis Guillermo Ibarra Ibarra”, Calz. México–Xochimilco No. 289, Col. Arenal de Guadalupe, Tlalpan, C.P. 14389 Ciudad de México, Mexico; ^3^Subdirección de Medicina del Deporte, Instituto Nacional de Rehabilitación “Luis Guillermo Ibarra Ibarra”, Calz. México–Xochimilco No. 289, Col. Arenal de Guadalupe, Tlalpan, C.P. 14389 Ciudad de México, Mexico; ^4^LAREMUS, Sección Bioelectrónica, Departamento de Ingeniería Eléctrica, Centro de Investigación y Estudios Avanzados del Instituto Politécnico Nacional, Av. Instituto Politécnico Nacional 2508, Col. San Pedro Zacatenco, Gustavo A. Madero, C.P. 07360 Ciudad de México, Mexico

## Abstract

Diabetic skin manifestations, previous to ulcers and wounds, are not highly accounted as part of diagnosis even when they represent the first symptom of vascular damage and are present in up to 70% of patients with diabetes mellitus type II. Here, an application for skin macules characterization based on a three-stage segmentation and characterization algorithm used to classify vascular, petechiae, trophic changes, and trauma macules from digital photographs of the lower limbs is presented. First, in order to find the *skin region*, a logical multiplication is performed on two skin masks obtained from color space transformations; dynamic thresholds are stabilised to self-adjust to a variety of skin tones. Then, in order to locate the *lesion region*, illumination enhancement is performed using a chromatic model color space, followed by a principal component analysis gray-scale transformation. Finally, characteristics of each type of macule are considered and classified; *morphologic properties* (area, axes, perimeter, and solidity), *intensity properties*, and a set of *shade indices* (red, green, blue, and brown) are proposed as a measure to obviate skin color differences among subjects. The values calculated show differences between macules with a statistical significance, which agree with the physician's diagnosis. Later, macule properties are fed to an artificial neural network classifier, which proved a 97.5% accuracy, to differentiate between them. Characterization is useful in order to track macule changes and development along time, provides meaningful information to provide early treatments, and offers support in the prevention of amputations due to diabetic feet. A graphical user interface was designed to show the properties of the macules; this application could be the background of a future *Diagnosis Assistance Tool* for educational (i.e., untrained physicians) and preventive assistance technology purposes.

## 1. Introduction

Diabetes is a rapidly growing chronic disease with a 20% prevalence and which is catalogued as a noncommunicable disease [1]. Diabetes mellitus type II is characterized by insulin resistance. Insulin is a hormone that helps deliver glucose to cells, e.g., to muscular cells where it is metabolized as energy [2]. Insulin resistance is a sign of diabetes development. In this process, which is called hyperglycemia, glucose is not delivered to the cells and builds up in the body.

According to the 2014 diabetes report from the World Health Organization (WHO) [3], in the world there are 422 million people living with diabetes. In developing countries, the prevalence is increasing. In Mexico, the Health Department via the 2016 National Health and Nutrition Survey (ENSANUT 2016) [4] reported that 9.4% of Mexican adults (i.e., 6.5 millions) have been diagnosed with diabetes. However, in 2017, the International Diabetes Federation (IDF) [5] reported that there are 12 millions of Mexican adults living with diabetes, but 37.5% are not aware that they have this disease.

Comorbidities such as obesity, hypertension, and dyslipidemia, among others, are precipitating factors to develop diabetes [6]. Even more, when these comorbidities are present along with diabetes, a rapid deterioration of body functions could arise and persist; diabetic retinopathy and diabetic foot [4] can cause blindness or lead to amputations which lead to disabilities.

Diabetes is associated, in the long-term, with degenerative processes that affect the cardiovascular and nervous system, as well as the eyes and skin [7]. From 30 to 70% of patients with diabetes develop skin problems [7, 8]. Neuropathy, microangiopathy, and macroangiopathy are the main predisposing factors for diabetic foot. Their evolution leads to blood flow reduction and ischemia, structural and functional damage, and an overloaded extremity due to the lack of sensitivity; all these put the foot at risk. Moreover, just adding up anything like a simple trauma or an infection could lead to ulcers, lesions, and even necrosis [9].

Although microangiopathy and macroangiopathy are major contributors to complications like skin lesions or diabetic foot, metabolic disruptions also have a significant direct effect, especially in alterations of the skin [7]. Some of these manifestations are called macules [10], which are defined as a flat, distinct, and discolored area of skin. Other manifestations may include lack of body hair, yellowish coloration, callous formation, onychomycosis, foot and toe deformation, and others [7, 8]. Even though macules occur commonly, they are not taken into account as a diagnose element [11, 12], nor are they registered as information that could lead to an early diabetic foot diagnosis [12, 13].

Relevantly, microangiopathy and macroangiopathy are also the cause of most skin manifestations found in patients with diabetes mellitus who have not been diagnosed with diabetic foot [14].

In the case of diabetes mellitus, skin manifestations have not been accounted as an important aspect of the disease [15]. There is a high prevalence of skin disorders among these patients as a matter of fact, and various authors report that these disorders are present in ∼70% of their patients [14].

Kiziltan et al. [14] state that diabetic dermopathy is more common on patients with neuropathy or large vessel disease; also, they report it as frequently present in patients with signs and symptoms of polyneuropathy. Pavicic and Korting [16] report that peripheral arterial obstructive disease (PAOD) is up to 6 times more frequent in patients with diabetes and PAOD, neuropathy, and macroangiopathy are key pathophysiologic factors in its development.

Several related studies report that 73% [15] to 80% [17] of the sampled patients present skin lesions or changes. Diabetic dermopathy always comes first as the most common skin manifestation in patients with diabetes. Pavicic and Korting [16] also state that the increasing duration of the disease rise the possibility of skin involvement; 45% of patients suffering diabetes for more than 20 years developed a peripheral vascular disease, and 75–82.1% presented xerosis, which could cause skin tears [16].

Any change in skin pigmentation is called a *macule*. Macules can be erythematosus (originated by blood vessels dilation or formation of new vessels), pigmentosae (which can be hyperpigmented, hypopigmented, or achromatic), or artificial, among others. Vascular macules occur as a secondary reaction, e.g., to medication, due to peripheral venous insufficiency or trauma [18].

A *vascular macule* is the one originated from a micro- or macrovascular problem, where the vessels underneath the skin are affected. These macules are rounded and are of reddish to brown color; normally, they present a diameter of 1 cm, but they can be smaller. *Petechiae* are very small (the size of a pinhead), reddish, rounded spots that appear on the shins and usually are a secondary effect of treatment with acetylsalicylic acid. *Macules due to trophic changes* are present when the patient has chronic venous insufficiency. They are darker patches of skin, have a larger area than other macules, and appear mainly in the ankles and shins. *Macules due to trauma* are the evidence (different than a scar or scab) of a traumatic event such as a hit in the shin. They are of brownish color, and the shape varies according to the trauma presented. This macule lingers in the skin of the patient with diabetes for a longer period of time than it would on a healthy patient.

All these skin manifestations are present previous to a diabetic foot diagnosis; patients can present them all at the same time, and they are generally overlaid. These macules appear in different parts of the leg and have large areas with undefined borders. Their localization and ulterior segmentation represent a challenging task, but the results can eventually be used as a tool for macule characterization, foot health prognosis, and even for amputation risk assessment.

Regarding the algorithms for image processing, these types of macules are not evaluated or processed until they become lesions or ulcers [19]. Computer aid diagnosis has been used in skin lesions for dermatology and dermoscopy (e.g., carcinomas and melanomas) [20–22] by means of support vector machines [21], support vector classification [20], or seeded region growing [22], but not in the prevention of diabetic foot development. Generally, tools for assessing skin problems due to diabetes mellitus type 2 are focused on advanced lesions and use questionnaires [18] that evaluate lesions like ingrown toenails, ulcers, calluses, or fissures, which take place after the diabetic foot diagnosis.

In this paper, we present the design of a graphical user interface (GUI) developed in Matlab® as an application for the characterization of skin macules. The GUI is based on a segmentation algorithm that applies image-processing techniques in order to find the region of interest (ROI) and characterize the macules present in images of the leg and foot of patients with diabetes mellitus type 2. We also present a statistical study of the calculated properties and a classifier of the 4 types of macules.

## 2. Materials and Methods

**Figure 1:**
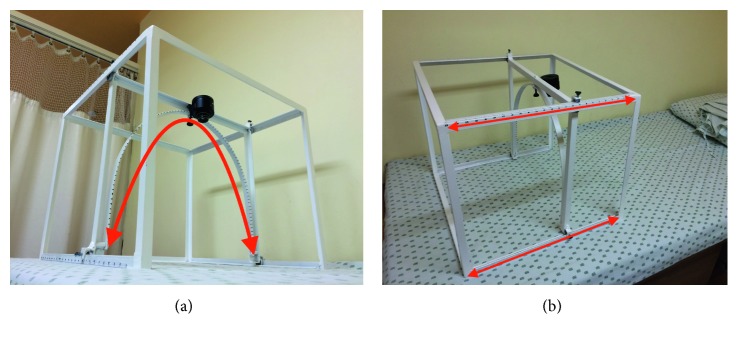
Description of WIAS. The camera is located on top of the device, and it can be moved along the arc; also, the frame can be slid horizontally. (a) Bottom view of the device. The arrow shows how the camera slides on the arc. (b) Top view of the device. The arrows show how the frame slides.

The first step was to acquire color digital photographs of skin macules “*skin images*” from the lower limb. For this purpose, a device called Wireless Image Acquisition System (WIAS) [23] was used. The device included a digital wireless camera (Sony DCS–QX100, 18MP), which provided an RGB image (Figure 1). Zoom and flash were never used in order to avoid changes in resolution or capturing bright areas, respectively. Changes in area, shape, and coloration of macules were document by the *skin images*.

The macules studied in this work were vascular macules, petechiae, macules due to trophic changes, and macules due to trauma. The study was performed at the Cardiac Rehabilitation Service of a National Institute in Mexico City. *Skin images* were processed using the Image Processing Toolbox of Matlab®. They were taken from 19 Mexican patients diagnosed with diabetes mellitus type II, but not yet with diabetic foot, who gave their signed informed consent.

Segmentation and characterization were performed through a proposed 3-stage image-processing algorithm, as described below:

**Stage 1 .**

(*skin region*). Skin identification in the color photography.

**Stage 2 .**

(*Skin region*-*lesion region*). Identification of possible lesions.

**Stage 3 .**

(*lesion region).* Characterization of macules.

### 2.1. Stage 1 (Skin Region)

The aim of using the WIAS device was to be able to acquire repeatable digital photographs from areas of interest form the lower limb, and these images were called *skin images*. The color *skin image* contained elements that were not of interest, e.g., the robe, the bed clothing, and other background components. So, the first objective was to segment the legs of the patient from it.

A color image can be transformed to different color spaces [24] (i.e., domain transformation) in order to enhance the characteristics of interest, i.e., the differences between skin and nonskin and the similarity among different skin tones. If we see the *skin image* as a matrix, size is determined by the resolution of the camera. The image has 3 levels of depth; each level corresponds to one RGB color matrix, and each cell in these matrices corresponds to a pixel, whose value is the level of intensity in 8 bits.

In Stage 1, the first step was to transform the image from RGB to HSV color space. RGB describes an image for the amount of red, green, and blue in it. HSV color space does the same but in terms of Hue, saturation, and value. The algorithm [25] is described by equation (1). The RGB values should be normalized to the range [0 1]:

(1) $$\begin{array}{c} \text{HSV}=\left\{\begin{array}{c} V=\max\left(R,G,B\right),\\ S=\frac{V-X}{V},\;\text{where}\;\;X=\min\left(R,G,B\right),\\ H=\left\{\begin{array}{cc} \frac{5+\left(\left(V-B\right)/\left(V-X\right)\right)}{6} & \text{for}\;\;R=V\;\;\mathrm{and}\;\;G=X,\\ \frac{1-\left(\left(V-G\right)/\left(V-X\right)\right)}{6} & \text{for}\;\;R=V\;\;\mathrm{and}\;\;G\neq X,\\ \frac{1+\left(\left(V-R\right)/\left(V-X\right)\right)}{6} & \text{for}\;\;G=V\;\;\mathrm{and}\;\;B=X,\\ \frac{3-\left(\left(V-B\right)/\left(V-X\right)\right)}{6} & \text{for}\;\;G=V\;\;\mathrm{and}\;\;B\neq X,\\ \frac{3+\left(\left(V-G\right)/\left(V-X\right)\right)}{6} & \text{for}\;\;R\neq V,\;\;G\neq V,\;\;\mathrm{and}\;\;R=X,\\ \frac{5-\left(\left(V-R\right)/\left(V-X\right)\right)}{6} & \text{for}\;\;R\neq V,\;\;G\neq V,\;\;\mathrm{and}\;\;R\neq X, \end{array}\right. \end{array}\right. \end{array}$$

where *V* represented the brightness value, *S* is the saturation, and *H* is the Hue matrix. The *R*, *G*, and *B* values had to be divided by 255 (e.g., *R* = *R*/255) in order to satisfy the normalization condition. The Hue matrix was selected (this property allowed the differentiation between ROIs and background), a fixed threshold was set, and an intensity range was determined to find a tone—set of values (equation (2)). This became the first skin mask:

(2) $$\begin{array}{c} \text{Skin}\;\;M\text{ask}\;\;1=\left\{\begin{array}{cc} \text{hue}\;\;\left(m,n\right), & 0.01\leq \text{intensity}\leq 0.1,\\ 0, & \text{else}. \end{array}\right. \end{array}$$

With the Skin Mask 1, it was not possible to identify a wide range of skin tones, so it was necessary to make the algorithm more robust. Therefore, a second color space transformation was applied using the YCbCr color space transformation matrix determined as follows [26]:

(3) $$\begin{array}{c} \left[\begin{array}{c} Y\\ \text{Cb}\\ \text{Cr} \end{array}\right]=\left[\begin{array}{c} 0\\ 128\\ 128 \end{array}\right]+\left[\begin{array}{ccc} 0.299 & 0.587 & 0.114\\ -0.169 & -0.331 & 0.500\\ 0.500 & -0.419 & -0.081 \end{array}\right]\left[\begin{array}{c} R\\ G\\ B \end{array}\right]. \end{array}$$

Then, a dynamic range was used. The histograms for the Cb and Cr matrix were calculated; then, these values were used to set dynamic limits in order to process different skin colors and tones in a wide range. This means that depending on the histogram values, the algorithm would adjust the threshold, so it would tune itself to the skin tone of the patient. The values found within the dynamic range outlined the second skin mask:

(4) $$\begin{array}{c} \text{Skin}\;\;\mathrm{Mask}\;\;2=\left\{\begin{array}{cc} 1, & \min\left(\text{Cb}\right)\leq \;\;\mathrm{Cb}\leq \text{mean}\;\;\left(\mathrm{Cb}\right)\;\;\mathrm{and}\;\;\mathrm{mean}\left(\text{Cr}\right)\leq \text{Cr}\leq \max\text{Cr},\\ 0, & \text{else}, \end{array}\right. \end{array}$$

where the limit values of Cb and Cr used in the equation changed for every skin tone found.

Then, in order to link the data from both color spaces, the HSV and YCbCr masks were added in an AND operation; this allowed for the resulting mask to work in a wide range of skin tones. This yielded a more robust algorithm for this stage and a more precise *skin region*.

### 2.2. Stage 2 (Lesion Region)

Once the *skin region* was segmented, *skin lesions* had to be identified. From the raw image in RGB color space, pixel values had to be amplified, so they became darker or lighter as they corresponded to healthy or damaged regions. For this purpose, the process described below was followed.

The color space *CIE 1976L*^*∗*^*a*^*∗*^*b*^*∗*^ was used to handle luminosity [27], in order to saturate the intensity values. This transformation was derived from the following equations [28]:

(5) $$\begin{array}{c} \left[\begin{array}{c} X\\ Y\\ Z \end{array}\right]=\left[\begin{array}{ccc} 0.4125 & 0.3576 & 0.1804\\ 0.2127 & 0.7152 & 0.0722\\ 0.0193 & 0.1192 & 0.9502 \end{array}\right]\left[\begin{array}{c} R\\ G\\ B \end{array}\right],\\ L=\left\{\begin{array}{cc} 116{\left(\frac{Y}{Y_{n}}\right)}^{1/3}-16, & \text{if}\;\;\;\frac{Y}{Y_{n}}>0.008856,\\ 903.3, & \text{else}, \end{array}\right.\\ a=500\left[\frac{X^{1/3}}{X_{n}}-\frac{Y^{1/3}}{Y_{n}}\right],\\ b=200\left[\frac{Y^{1/3}}{Y_{n}}-\frac{Z^{1/3}}{Z_{n}}\right]. \end{array}$$

**Figure 2:**
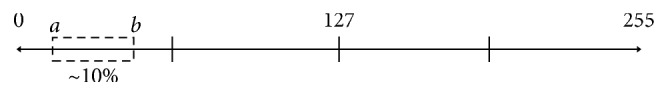
Selected PCA grayscale values. The values from *a* to *b* represented approximately 10% of the total and were the ones used to determine *lesion region*.

For this stage, the *L* matrix corresponded to luminosity from black to white and was the one selected [29]. The resulting saturated image was then reenhanced by converting it to grayscale using principal component analysis (PCA) [30]. *Lesion region* was calculated using the histogram of the PCA grayscale image, where a threshold was set to find the damaged areas. This threshold also shifted depending on the tones detected from the healthy and the lesioned skin, but it took approximately 10% of the values found in the image (Figure 2).

### 2.3. Stage 3 (Characterization)

Characterization of the damage in the lower limbs of the patients was performed in 2 stages:

(1) Data values of extracted features at the segmented *lesion region* were classified into 2 types: morphologic properties—area, major axis, minor axis, perimeter, solidity—and intensity properties—maximum intensity and minimum intensity.
(2) The *Shade Index* (ShI) was a parameter used to measure color variations from the RGB raw image. An equation was designed for each color, where equation (6) was used for the Shade Index Red (ShI_R_), equation (7) for the Shade Index Green (ShI_G_), and equation (8) for the Shade Index Blue (ShI_B_):
  (6) $$\begin{array}{c} {\text{ShI}}_{\text{R}}=\frac{\text{mean}\left(M_{\text{red}}\right)}{\text{mean}\left({\text{HS}}_{\text{red}}\right)}, \end{array}$$
  (7) $$\begin{array}{c} {\text{ShI}}_{\text{G}}=\frac{\text{mean}\left(M_{\text{green}}\right)}{\text{mean}\left({\text{HS}}_{\text{green}}\right)}, \end{array}$$
  (8) $$\begin{array}{c} {\text{ShI}}_{\text{B}}=\frac{\text{mean}\left(M_{\text{blue}}\right)}{\text{mean}\left({\text{HS}}_{\text{blue}}\right)}, \end{array}$$
  where *M*_red_ is the red component of the area inside the segmented macule, *M*_green_ is the green component of the area inside the segmented macule, and *M*_blue_ is the blue component of the area inside the segmented macule in RGB; HS_red_ is the red component of an area of healthy skin around the macule, HS_green_ is the green component of an area of healthy skin around the macule, and HS_blue_ is the blue component of an area of healthy skin around the macule, in RGB. Finally, a Shade Index Brown (ShI_BR_) (equation (9)) was used to identify brownish changes in the skin:
  (9) $$\begin{array}{c} {\text{ShI}}_{\text{BR}}=\frac{\text{mean}\left(M_{\text{red}}\right)+\text{mean}\left(M_{\text{blue}}\right)}{\text{mean}\left({\text{HS}}_{\text{red}}\right)+\text{mean}\left({\text{HS}}_{\text{blue}}\right)}. \end{array}$$

**Figure 3:**
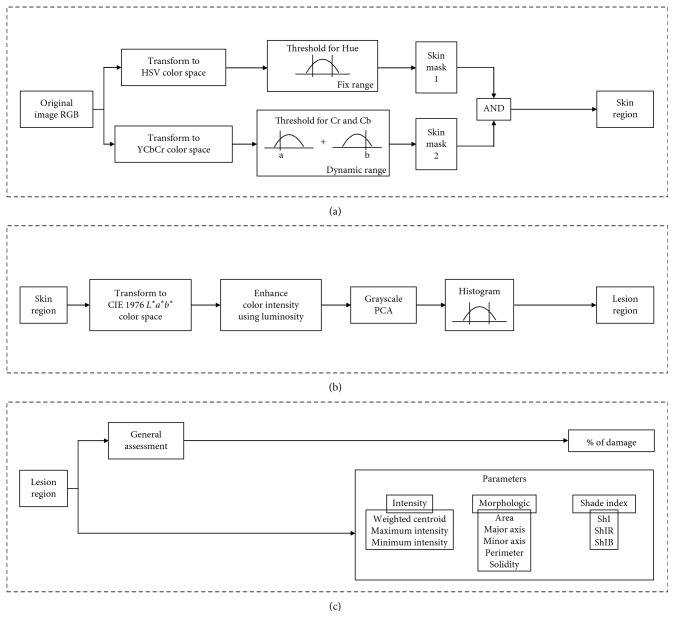
Three-stage algorithm: (a) Stage 1—processing from *skin image* to segment a *skin region* using the AND of two skin masks (via HSV and YCbCr color space transformation). (b) Stage 2—*lesion region* segmentation from the *skin region* by means of CIE 1976 *L*^*∗*^*a*^*∗*^*b*^*∗*^ transformation followed by luminosity enhancement and PCA grayscale transformation. (c) Stage 3—Characterization of the *lesion region*. Calculation of the parameters for *intensity*, *morphologic* and the *shade indices* for macules.

Figure 3 shows the flow diagram for the 3-stage algorithm for *skin* and *lesion region* segmentation in addition to the characterization feature.

It was necessary to find out if the differences were statistically significant among the values calculated for the extracted features in the algorithm for each type of macule. In order to validate this, Student's *t*-test was performed using SPSS v17 with a confidence interval of 95% (*p* < 0.05).

Also, a classifier was designed in order to identify each macule by means of building an artificial neural network and the feature vectors that characterize each of them. 60% of the data was used to train the network and 40% to test it.

## 3. Results and Discussion

The 3-stage image-processing algorithm reported in this paper is composed by segmentation of skin and its lesions, as well as the values of the features obtained from the *shade indices*.

Using the *skin images* acquired with the WAIS, the specialist classified the macules found in the patients as vascular and petechiae, due to trophic changes, or due to trauma macules. The results of image processing for the segmentation of *skin region* are shown in Results for Stage 1. Segmentation of *lesion region* is later displayed in Results for Stage 2, and the features of macule characterization are obtained and analyzed in Results for Stage 3.

### 3.1. Results for Stage 1 (Skin Region)

**Figure 4:**
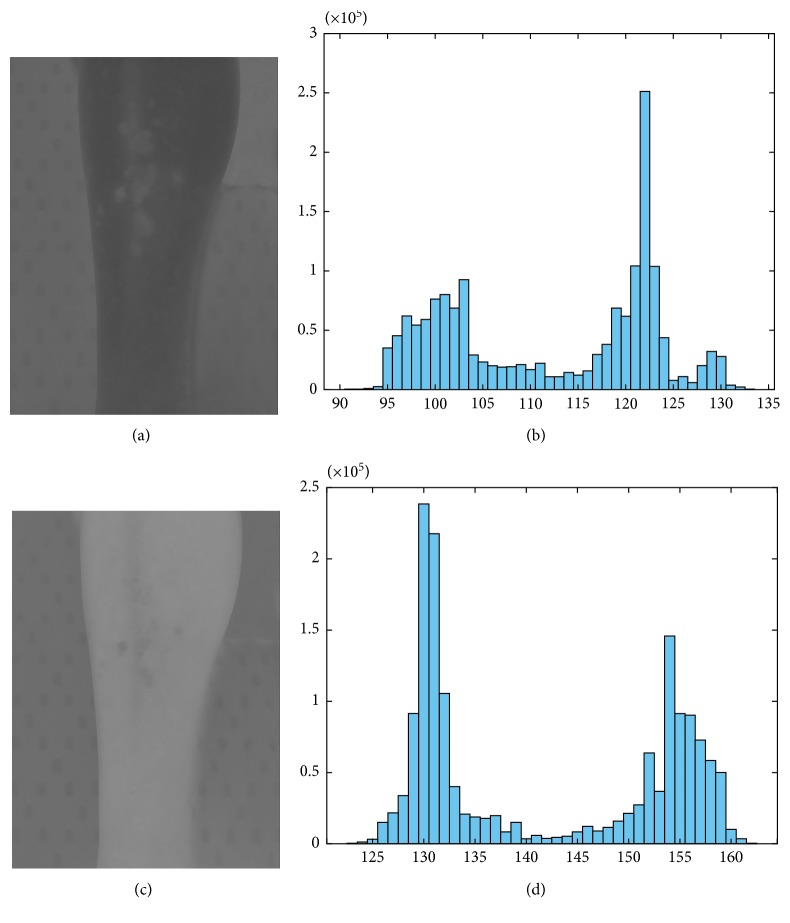
Histograms used to find the dynamic range: (a) image obtained for the Cb component from the YCbCr color space transformation; (b) Cb matrix histogram; (c) image obtained for the Cr component from the YCbCr color space transformation; (d) Cr matrix histogram.

An example of the histogram obtained after the YCbCr color space transformation used to find the dynamic range that self-adjusted to a wide variety of skin tones is shown in Figure 4. Mean value for each matrix fell in the valley of the histograms; the first section of Cb and the second section of Cr were selected in order to find the values that outlined the second skin mask.

**Figure 5:**
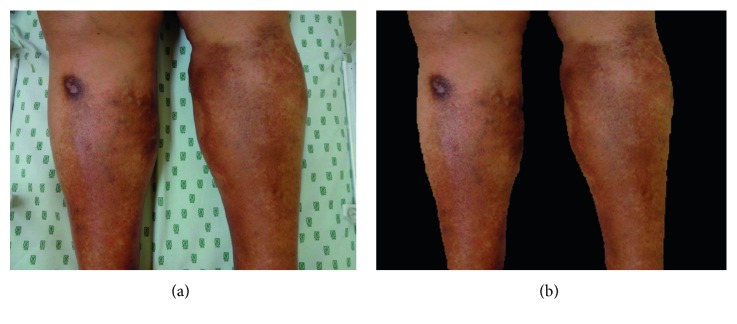
Stage 1 (*skin region*) segmentation example: (a) raw *skin image*; (b) segmented *skin region*.

*Skin region* was obtained from *skin image*, as shown in Figure 5. The background was eliminated with the intention of avoiding segmentation errors due to, e.g., the logo of the bed sheets or any object in the back.

### 3.2. Results for Stage 2 (Lesion Region)

**Figure 6:**
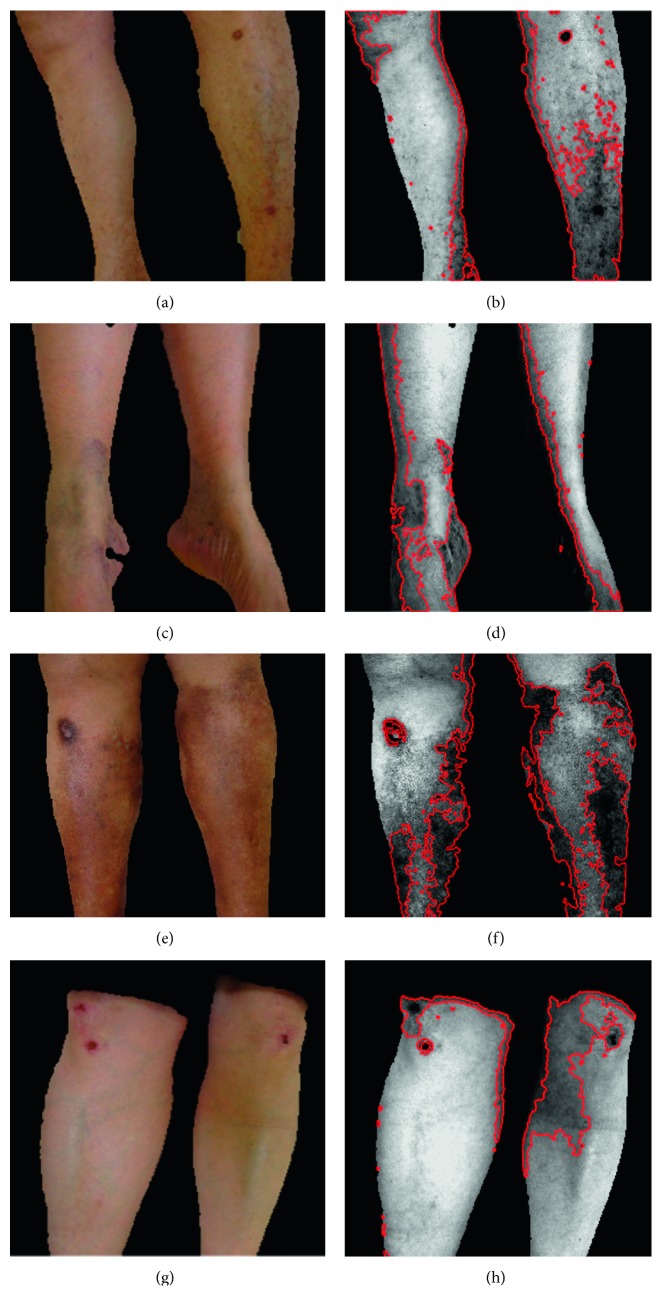
Example of segmentation of *skin regions* and *lesion regions* found in Stage 2 for four patients: (a) patient no. 1, *skin region*; (b) patient no. 1 shows 29% damage due to vascular damage and a trauma; (c) patient no. 2, *skin region*; (d) patient no. 2 shows 24% damage due to petechiae and vascular damage; (e) patient no. 3, *skin region*; (f) patient no. 3 shows 31% damage due to trophic changes, a trauma, and vascular damage; (g) patient no. 4, *skin region*; (h) patient no. 4 shows 21% damage due to traumatic lesions and vascular damage.

Figure 6 shows examples of different *lesion regions* (which include vascular, petechiae, trophic changes, and trauma macules) found in 4 patients. These images were the result of applying the novel proposed algorithm. From these examples, it was noticeable that some areas could be overseen in the RGB images, but after the processing enhancement with the *CIE 1976L*^*∗*^*a*^*∗*^*b*^*∗*^ color space transformation, the selection of the luminosity matrix, and the PCA gray-scale transformation, these hidden macules are now within the spectrum of the dynamic range selected from the histogram. From this stage, a general state of health of the extremity was calculated and displayed as percentage of damage (29% for patient no. 1, 24% for patient no. 2, 31% for patient no. 3, and 21% for patient no. 4).

### 3.3. Results for Stage 3 (Characterization of Features)

In order to characterize the macules (vascular, petechiae, due to trophic changes, or due to trauma), feature extraction for morphologic properties, intensity properties, and *Shade Indices* was performed in 82 macules obtained from the *lesion regions* found.  Table 1 shows the values obtained.

By means of statistical analysis, significant differences (*p* < 0.05) were found among the macules studied; these *p* values are shown in Table 2. According to it, petechiae and vascular macules can be differentiated through *morphologic properties* and *Shade Indices* (except ShI_B_). Differences between petechiae and macules due to trophic changes can be found comparing their *morphologic properties*; *morphologic properties* and *Shade Indices,* ShI_R_, were significantly different for petechiae and trauma macules. Vascular macules and those resulting from trophic changes can only be differentiated through their *morphologic properties*, while trauma macules can be differentiated comparing all properties expected for *solidity* and *minimum intensity*. Macules due to trophic changes and trauma can be differentiated using the *Shade Indices*: ShI_G_, ShI_B_, and ShI_BR_, and 4 other properties.

**Figure 7:**
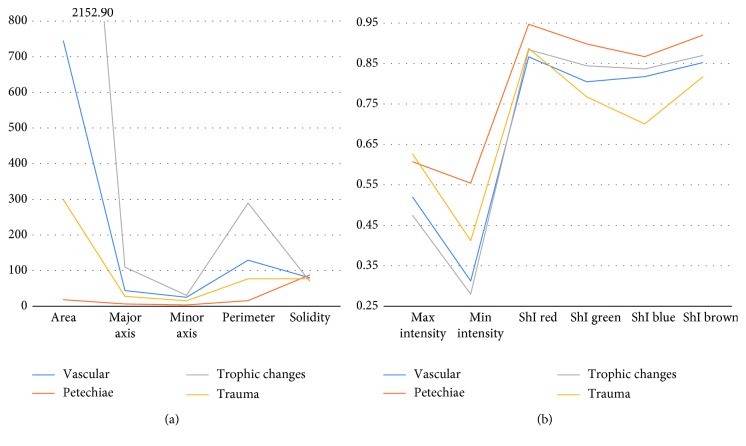
Average values of (a) morphologic properties and (b) intensity properties and shade indices.

The concatenation of properties, calculated for each macule evaluated, form the feature vector for each example. Figures 7(a) and 7(b) show the average value for each property and macule, or the average feature vector.

**Figure 8:**
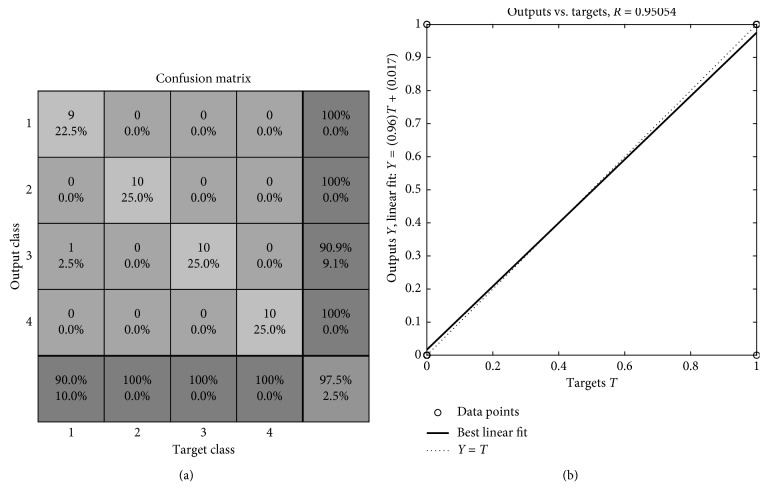
Classifier results. (a) Confusion matrix for the macule classifier. Class 1: vascular macule. Class 2: petechiae. Class 3: trophic changes. Class 4: trauma. (b) Linear regression that shows the relation between the elements of the network response and the corresponding targets.

So, in order to identify each macule, the proposed architecture is a feedforward backpropagation network with 2 hidden layers and 4 neurons per layer; the transfer functions are hyperbolic tangent sigmoid and logsigmoid. The training function updates weight and bias values according to the Levenberg–Marquardt optimization [31]. In order to train the classifier, an 11 × 40 matrix was built, where each type of macule yielded 10 examples; 60% of the data was used for the training, and the remaining 40% was used to test the network. The results were displayed through a confusion matrix (Figure 8(a)), where the coincidence between one of the 10 feature vectors and the target class was demonstrated. The correct identification of the data corresponded to 97.5%. A linear regression of the data (Figure 8(b)) shows the relation between the target data and the results obtained from the network, where *R* = 0.95054 indicates that the model was capable of identifying ∼95% of the segmented lesions.

### 3.4. Skin Macules Characterization (SMaC) Software

**Figure 9:**
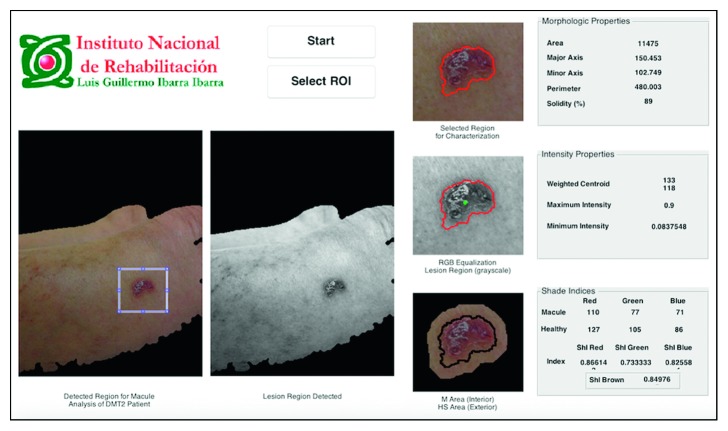
SMaC Software© for segmentation and characterization. This example shows the segmentation and characterization of a trauma macule.

A Matlab® GUI, called skin macules characterization (SMaC) Software©, showed the results of the 3-stage image-processing algorithm for segmentation of the skin macules, feature extraction, and characterization of 4 types of macules: vascular, petechiae, trophic changes, and trauma, as seen in Figure 9. This software was registered at the National Copyright Institution of Mexico (INDAutor, no. 03–2017–071912253000–01).

Macule images need special processing algorithms as they are very peculiar and present different features depending on the patient. There are no algorithms reported to address this particular problem. Moreover, because of the wide range of human skin color tones, the major challenge to overcome was to find the macules in spite of the changes in illumination among the *skin images*.

Color space transformations became a useful tool to find different views of the image that allowed enhancing characteristics that were convenient to solve the segmentation problem. In the HSV color space transformation, the Hue values selected showed a good performance with medium skin colors, but it depended of the light in the room. In order to address this situation, a second color space transformation was applied (YCbCr). This color space extracted red and blue components from the image; since macule color varies from red to brownish, these components became very helpful for macule location and segmentation.

In this case, a fix range for the Cb and Cr values was not useful, even when it is the method of choice in the literature [26, 28] because it limits the variety of human skin tones detected to a small selection. However, the dynamic range proposed in this paper allowed the algorithm to adjust to a wide range of skin tones, which increases its usefulness meaningfully. The minimum and maximum values taken from the Cb and Cr matrices represented the illumination range of the image. Histogram values allowed the algorithm to self-tune to the specific image and hence to the specific skin tone and illumination, maintaining the simplicity and efficiency of the algorithm without adding the computational cost of neural networks. The position of the camera can be adjusted using the *WIAS* device in order to avoid areas with too much brightness or intense illumination.

A normal grayscale transformation was not useful for skin segmentation because it equalizes the distribution of gray levels, which is counterproductive for this scenario. On the contrary, PCA generates an image in gray levels within the limit values of the histogram of one specific image every time, it gets rid of the healthy tissue in the image and keeps the sections with clear manifestations of saturation, and these sections are classified and selected as lesioned skin. This technique helps take advantage of the illumination enhancements achieved by the *CIE 1976L*^*∗*^*a*^*∗*^*b*^*∗*^ transformation used in Stage 2. And, again, to set thresholds and ranges through histogram, values allow the algorithm to adjust to the particularities of the *lesion region* found without the need to use more complex processing in order to classify between healthy and lesioned skin. So, this algorithm is a simple and efficient solution for processing macules, which can have multiple applications.

Ideally, in an image identification process posterior to a segmentation, it is important to have a feature or property that allows distinguishing between classes of a group of data. This can be complicated depending on the characteristics of what is being identified in the image, so quantitative parameters are preferred to guarantee a more robust result.

Therefore, the macules measured were characterized using *morphologic properties*, to define shape and geometry; *intensity properties*, to establish maximum and minimum values of the pixels within the lesion and to separate them from the healthy skin; and the proposed *Shade Indices*, to identify lesions by color tone. During the development of the characterization (Stage 3), it became evident that, e.g., color red (in the RGB image) did not look the same in every skin tone or even with different illumination, so the reference value could not be fixed. The solution to overcome these problems was to come up with a novel set of *Shade Indices*, where the healthy skin around the macule was used as a reference for color tone shifts. On the contrary, ShI_R_ and ShI_BR_ turned out to be the indices that helped differentiating the most between the macules studied.

*Morphologic properties* are features of the macule in which it was possible to point out geometric and shape variations among most of them. *Intensity properties* did not seem to have a considerable input for classification of data since their *p* values were significant in less than 40% of the relations studied.

In general, from this analysis, it was comprehensible how complex the problem was since different kinds of macules were present at the same time and, even more, they were overlaid. From the image segmentation and processing point of view, there was a high difficulty to isolate the *lesion region* to provide an accurate assessment. Nevertheless, with the macule properties chosen and calculated, it was possible to classify each type of macule with 97.5% of accuracy.

With the use of the SMaC Software© characterization and latter classification, the macules of patients with diabetes can be measured and tracked along the development of the disease in order to prevent further disabilities and comorbidities. The use of this software can be especially beneficial for those physicians who do not have specialized training or enough expertise to identify specific macules; it can also be used as an educational tool. The perception of the importance on skin manifestations that appear previous to ulcers or amputations must be changed since they seem to be the first symptom of endothelial decay and vascular damage which lead to worse symptoms of diabetic foot and, eventually, to amputation. From the clinical perspective, the origin of skin and limb damage is multifactorial, but it relates mainly to endothelial decay.

In the future, we aim to turn this GUI Software into a *Diagnosis Assistance Tool*, which would include clinical variables and other diabetic foot manifestations in order to gather enough data to eventually form a database of patients with diabetes, for preventive purposes.

## 4. Conclusion

Nowadays, lower limb skin manifestations are not taken into account in the general evaluation of the state and development of diabetes mellitus type II, even when they have an underlying vascular origin. This paper presents the application of an algorithm for the segmentation, characterization, and classification of skin manifestations from photographic images and the identification of them in the lower limbs of diabetic patients. An efficient algorithm for image processing of skin macules characterization performed by means of extracting *morphologic* and *intensity properties* is proposed, along to a new set of *Shade Indices* used to assess color shifts in different skin tones. From the three sets of features, *morphologic properties* and *Shade Indices* resulted statistically significant in order to differentiate among macules of various origins. The indices described here are a new way to assess changes in color for different skin tones, which increase the usefulness of the application. The properties extracted are used as feature vectors for the input of a classification network which resulted in a 97.5% accuracy for the 4 types of macules studied in this paper: vascular, petechiae, trophic changes, and trauma. The SMaC Software© was designed to bring the proposed algorithm as a tool for the physician in order to aid in the identification and assessment of skin lesions in the lower limbs of patients with diabetes.

## Figures and Tables

**Table 1:** Characteristic feature values found for vascular, petechiae, trophic changes, and trauma macules.

Macule	Vascular (*n*=47)	Petechiae (*n*=10)	Due to trophic changes (*n*=10)	Due to trauma (*n*=15)
*Morphologic properties*				
Area (pixels)	1010.29 ± 1893.99	18.50 ± 5.99	2152.90 ± 1541.85	2133.60 ± 3684.09
Major axis (pixels)	46.97 ± 48.96	6.48 ± 1.60	109.97 ± 54.13	59.08 ± 63.30
Minor axis (pixels)	23.76 ± 17.75	3.88 ± 1.08	30.67 ± 13.49	29.85 ± 24.87
Perimeter (pixels)	143.90 ± 154.46	15.83 ± 4.76	289.46 ± 116.72	166.16 ± 173.27
Solidity (%)	72.56 ± 12.76	88.00 ± 12.97	70.40 ± 11.96	79.87 ± 9.31
*Intensity properties*				
Maximum intensity	0.55 ± 0.18	0.61 ± 0.14	0.47 ± 0.16	0.62 ± 0.13
Minimum intensity	0.35 ± 0.18	0.55 ± 0.14	0.28 ± 0.19	0.31 ± 0.21
*Shade index*				
ShI_R_	0.90 ± 0.06	0.95 ± 0.03	0.88 ± 0.07	0.86 ± 0.08
ShI_G_	0.84 ± 0.08	0.90 ± 0.07	0.84 ± 0.09	0.74 ± 0.14
ShI_B_	0.82 ± 0.11	0.87 ± 0.09	0.84 ± 0.08	0.68 ± 0.15
ShI_BR_	0.87 ± 0.11	0.92 ± 0.04	0.87 ± 0.07	0.79 ± 0.10

**Table 2:** *p* values for the differences among features data distribution for each macule relation.

Macule	Petechiae vs vascular	Petechiae vs trophic changes	Petechiae vs trauma	Vascular vs trophic changes	Vascular vs trauma	Trophic changes vs trauma
*Morphologic properties*						
Area	0.0001^*∗*^	0.0001^*∗*^	0.043^*∗*^	0.0001^*∗*^	0.002^*∗*^	0.986
Major axis	0.0001^*∗*^	0.0001^*∗*^	0.006^*∗*^	0.0001^*∗*^	0.012^*∗*^	0.043^*∗*^
Minor axis	0.0001^*∗*^	0.0001^*∗*^	0.001^*∗*^	0.0001^*∗*^	0.003^*∗*^	0.916
Perimeter	0.0001^*∗*^	0.0001^*∗*^	0.005^*∗*^	0.0001^*∗*^	0.011^*∗*^	0.044^*∗*^
Solidity	0.003^*∗*^	0.005^*∗*^	0.080	0.457	0.054	0.036^*∗*^
*Intensity properties*						
Maximum intensity	0.129	0.059	0.776	0.406	0.026^*∗*^	0.015^*∗*^
Minimum intensity	0.0001^*∗*^	0.002^*∗*^	0.002^*∗*^	0.343	0.632	0.682
*Shade index*						
ShI_R_	0.0001^*∗*^	0.023^*∗*^	0.0001^*∗*^	0.598	0.038^*∗*^	0.373
ShI_G_	0.024^*∗*^	0.157	0.0001^*∗*^	0.668	0.004^*∗*^	0.035^*∗*^
ShI_B_	0.104	0.435	0.0001^*∗*^	0.448	0.001^*∗*^	0.003^*∗*^
ShI_BR_	0.004^*∗*^	0.072	0.0001^*∗*^	0.899	0.001^*∗*^	0.035^*∗*^

^*∗*^Statistically significant *p* values (*p* < 0.05).

## Data Availability

Photographic images of macules at the lower limbs of patients with diabetes used to support the findings of this study are restricted by our Institutional Ethics and Research Review Board in order to protect patients' privacy. The data may be released upon petition to Research Review Board who establishes the criteria to access confidential data.
